# Masticatory Muscles Activation and TMJ Space During Asymmetrically Loaded Jaw Closing

**DOI:** 10.1007/s10439-023-03424-6

**Published:** 2024-01-12

**Authors:** Lea Angst, Jan Harm Koolstra, Daniel Wiedemeier, Rachel M. Van Sluijs, Anina M. Pulfer, Luigi M. Gallo, Vera Colombo

**Affiliations:** 1 Clinic of Masticatory Disorders, Centre of Dental Medicine, University of Zurich, Plattenstrasse 11, 8032 Zurich, Switzerland; 2 Department of Functional Anatomy, Academic Centre for Dentistry Amsterdam (ACTA), Research Institute MOVE, University of Amsterdam and VU University, Amsterdam, The Netherlands; 3 Statistical Services, Center of Dental Medicine, University of Zurich, Zurich, Switzerland

**Keywords:** Biomechanics, Temporomandibular joint, Masseter muscle, Temporalis muscle, Minimization of joint load, Minimization of muscle effort

## Abstract

Masticatory muscle activation and temporomandibular joint (TMJ) load generated during asymmetrically loaded jaw closing are largely unknown. Two different strategies were developed to explain how the central nervous system (CNS) generates muscle activation patterns during motion: minimization of joint load (MJL) vs. minimization of muscle effort (MME). The aim of the present study was to investigate, experimentally, the neuromuscular strategy selected by the CNS to coordinate jaw closing in reaction to the application of an external asymmetric load. Masticatory muscle activation was measured with electromyography (EMG) and the minimum intra-articular distance (MID) was assessed by dynamic stereometry to infer joint loading. Ten healthy subjects performed jaw-closing movements against an asymmetric mandibular load set from 0.0 to 2.0 kg in 0.5-kg steps. Recordings were analyzed by exploratory and graphical statistical tools. Moreover, the observed differences in MID and EMG among the various mandibular loads were tested using non-parametric tests for repeated measures data. The ipsilateral-contralateral differences in MID and EMG of the anterior temporalis showed a significant increase (*p* < 0.001, *p* = 0.01) with increasing asymmetrical load with both joints being most heavily loaded at 1 kg. EMG signals of the masseter did not change significantly with increasing load. This study is the first to have analyzed the changes in the TMJ intra-articular space during asymmetrically loaded jaw-closing movements, not only three dimensionally and dynamically, but also combined with EMG. Asymmetrical load affected the TMJ space and masticatory muscle activation patterns, primarily resulting in an increased activation of the anterior temporalis muscle. This might suggest the involvement of a control mechanism to protect the joints from overloading. However, the results do not fully support the hypothesis of MJL nor the MME strategy.

## Introduction

Complex mandibular movements with six degrees of freedom require sophisticated neuromuscular activation by higher centers of the brain. Investigations by magnetic resonance imaging and transcranial magnetic stimulation showed that elemental and learned orofacial tasks, such as jaw closing, are most notably coordinated by the face primary motor area and primary somatosensory area [1, 2]. These centers integrate sensomotoric feedback with experiences and expectations [3]. A biologically relevant goal, such as minimization of joint load (MJL) or minimization of muscular effort (MME), might be crucial for the balance of muscle activation and joint loading [4–6]. Movements with the same ultimate goal, i.e., jaw closing, can be achieved by different muscle recruitment patterns (*overdetermination*) and thus, the metabolic costs and loading of the system might differ widely [7].

The idea of optimization strategies has roots in evolutionary biology. Important research was provided by W.A. Sparrow and colleagues on metabolic energy expenditure [7, 8]. Following this concept, different optimization objectives have been proposed in various studies on the human masticatory system [6, 9–12]. Several studies documented the optimization strategies during isometric muscle contractions, such as unilateral and bilateral static biting tasks. One study revealed that the medial pterygoid was the most active muscle for all biting directions and that biting with accentuated horizontal force components provoked the largest joint reaction force [13]. Another study showed that the individual variability in the patterns and magnitudes of muscle forces were considerable [14]. A third study pointed out that in terms of optimization strategies, large inter-individual differences were observed regarding the consistency of masticatory muscle forces exerted during static biting with objectives of MJL or MME strategies [4]. The shape of the articular eminence in healthy subjects, however, seems to be linked to MJL [5]. Although several studies investigated CNS optimization strategies during static biting without a conclusive understanding of neuromuscular control strategies [4, 5], to date, no evidence is found in literature concerning the coordinative role of MJL or MME during mandibular movements.

The present study focused on jaw-closing movement, which involves concentric contraction. The authors expected a more conclusive picture by investigating this dynamic set-up. A study by van Eijden et al. showed that subjects tend to choose the same unique, reproducible muscle activation pattern for a particular task [15]. This activation pattern is probably learned during development and selected for its biological benefits over other possible patterns. As suggested by Osborn and Baragar, it may be possible that muscle activation is altered due to peripheral sensory feedback in order to avoid a system overload and protect the joint from damage [11]. TMJ loading depends not only on the forces generated by jaw muscles but also on loading direction [16]. As the results of the former study were based on static biting and not on mandibular movement, the investigation of how changes of this particular set-up could influence the results are of great interest.

The aim of the present study was to analyze the effect of an increasing external load (up to 2.0 kg) during jaw closing on (i) TMJ load, reflected by the variations of minimum intra-articular space, (ii) anterior temporalis muscle activation, and (iii) masseter muscle activation, by means of dynamic stereometry and electromyography. The asymmetric load set-up was chosen to assess the response of the CNS to a perturbation to its habitual unloaded symmetric closing pattern and to observe its control strategy.

The authors hypothesize that, if during asymmetrically loaded closing movements, the muscle coordination patterns are driven by the MME goal**,** an asymmetric joint loading would be observed. Alternatively, if they are driven by the MJL goal, then asymmetric muscle activity should be observed. The null hypothesis is that the MID and EMG of masseter and anterior temporalis muscles are not changing with increasing unilateral load both ipsilaterally and contralaterally to the load application.

## Methods

### Subjects

Ten healthy subjects (7 women, 5 men; mean age 30 ± 7 years) were recruited among students attending the University of Zurich. The sample size was chosen based on a previous study [17]. The subjects were recruited according to following inclusion criteria: age between 20 and 40 years; willingness to participate in the study. Prior to definitive inclusion, subjects were screened and excluded if found to have orofacial pain and/or temporomandibular disorders (assessed by calibrated examiners according to Axis l of the Diagnostic Criteria for DC/TMD [18]); symptoms or history of disorders affecting the neuromuscular system; chronic pain conditions in other parts of the body; and a habitual intake of drug or alcohol abuse influencing the CNS. In women of childbearing age, a pregnancy test, approved by the Ethics committee, was performed before the MR session and pregnant women were denied participation. Before committing to the study, the subjects provided signed, informed consent.

The study followed the Declaration of Helsinki regarding medical protocols and ethics and the Ethics Committee of the State of Zurich approved the study (KEK-ZH-No 2018-01424).

### Experimental Set-Up

**Fig. 1:**
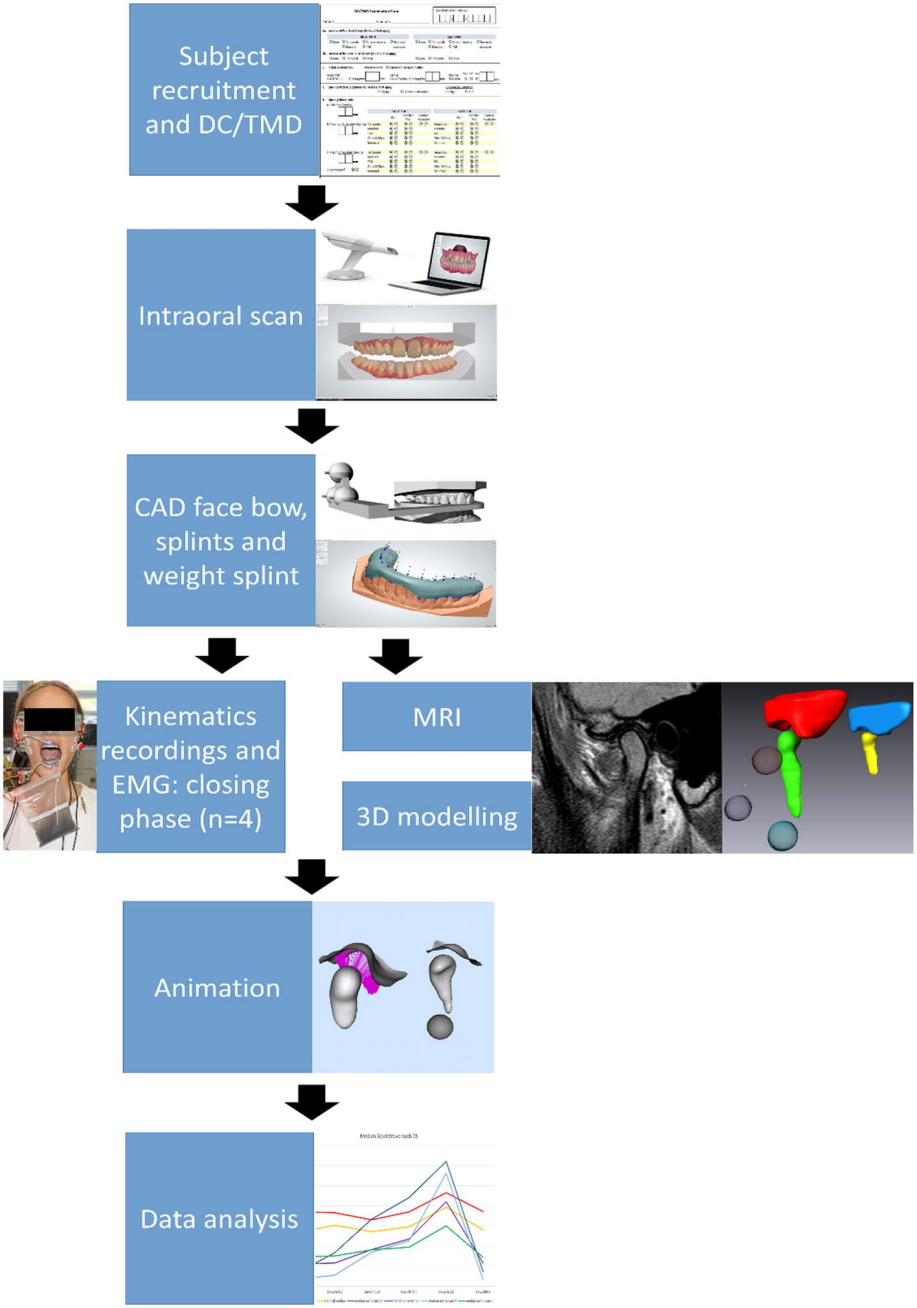
Study protocol flowchart. *DC/TMD* diagnostic criteria for temporomandibular disorders, *CAD* computer-aided design, *MRI* magnetic resonance imaging, *EMG* electromyography.

The study followed the protocol shown in Figure 1. TMJ load measurements could not be performed directly *in vivo*, therefore an indirect approach was used. Dynamic stereometry is a non-invasive research method producing subject-specific animations of anatomic three-dimensional models by combining imaging and motion-tracking data [19, 20]. This method allows for a quantitative and dynamic measurement of the intra-articular space during mandibular motion and can indirectly be used as a measure of the areas undergoing the highest stresses in the joint soft tissues [21, 22]. First, virtual 3D models of the joint articular surfaces (mandibular condyle and temporal fossa) were obtained by tracing the bone contours acquired by MR imaging and then creating two triangular mesh objects. Segmentations of the anatomy were performed by the same experienced operator for all study participants. The polygon type and mesh density were identical in all models to ensure comparable inter-individual results. Second, mandibular movements were tracked using a custom-built optoelectronic device (OPTIS). The system consists of three linear cameras with cylindrical lenses (dimensions: 25 × 50 mm, focal length: 75 mm, Edmund Optics Ltd, York, UK) and charge-coupled device sensors (CCD arrays of 2048 elements, 26.624 mm length, 220 Hz, spatial resolution ≤ 5 μm) fixed on a metal bar. The cameras track the position of two triangular target frames (TTFs), each formed by a triplet of light-emitting diodes (LEDs), glued to the subject’s upper and lower dental arches by means of individualized splints. The signals from the CCDs are sent via a digital signal processor (DSP) to a Windows^®^-based computer over TCP/IP protocol, transformed into a stream of Cartesian coordinates and written on mass storage media [23]. A face bow (FB) was used to register imaging and movement data into a common coordinate system. The FB was designed subject specifically and provided a bite plate to allow intercuspation and carried three non-collinear spheres with MR contrast medium and three non-collinear LEDs in calibrated geometry [19, 20, 22, 24, 25]. Subjects were requested to wear the FB during the entire MR imaging session and at the beginning of the kinematic session (during a reference recording), while biting on it, in order to allow for the combination of datasets within the same coordinate system.

Electromyography (EMG) was used to measure the bilateral electrical potentials in the masseter and anterior temporalis muscles during the jaw-closing movement. For this purpose, pairs of self-adhesive pre-gelled disposable bipolar surface electrodes with a recording surface of 95 mm^2^ were placed along the estimated main fiber direction of the muscles and 2 cm apart from each other in accordance with the SENIAM guidelines (Neuroline Type 720 00-S/25, Ambu, Ballerup, Denmark) [26]. EMG was acquired with a customized two-channel recorder with a sampling frequency of 2 kHz, 20–400 Hz bandpass filter, and a 4000 × gain [27, 28].

### Data Collection

Prior to the first experimental session, a digital three-dimensional impression of the dentition was obtained by means of a commercial intra-oral scanner (TRIOS™, 3Shape, DK-Copenhagen; resolution 6.79–12.58 µm) [29]. Based on the scans, a weight splint for asymmetrical mandibular loading and a face bow were custom designed and 3D printed (Rhino 5®; McNeel Inc., Seattle WA, USA; https://www.rhino3d.com; TRIOS; 3Shape appliance designer™, Copenhagen, Denmark; Objet Eden 260 V™; Stratasys, Eden Prairie MN, USA).

At the second appointment, an MRI scan perpendicular to the condylar long axis was obtained for both TMJs with image stack dimensions of 160 × 160 × 75 mm and voxel size 0.9 × 0.9 × 0.9 mm^3^ (Gyroscan ACS-II R, Philips Medical Systems, the Netherlands; 1.5 Tesla). The anatomic surfaces of the condyle and fossa were subsequently reconstructed three dimensionally from the MR images (Amira™ v. 6, FEI, Hillsboro OR, US).

**Fig. 2:**
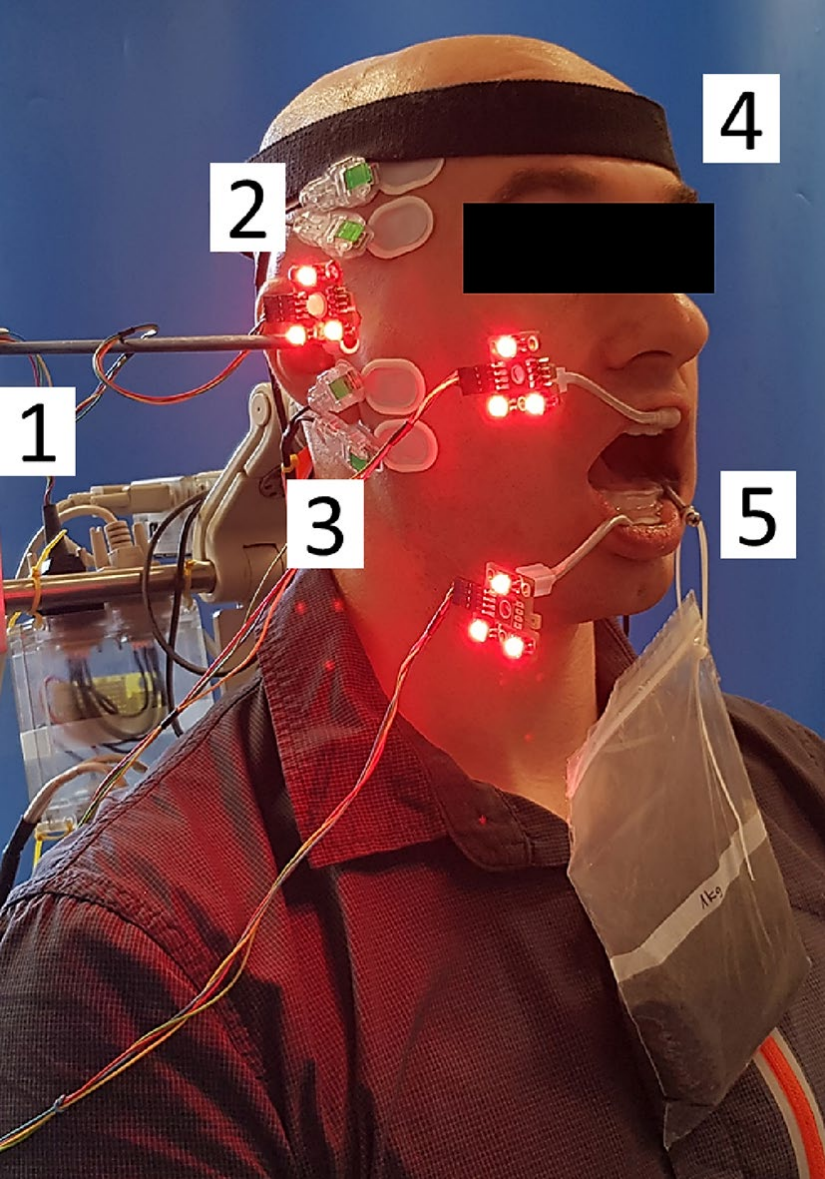
Subject performing a closing movement with weight splint in place. An electromyogram (1) was recorded from surface electrodes placed on the skin overlaying M. temporalis anterior (2) and M. masseter (3). The subject’s head was secured (4) in order to keep an upright position during the closing movement against the resistance of the asymmetric load (5).

During the third appointment, surface electrodes for the EMG recordings were placed on the subjects’ skin overlaying the left mastoid process. Prior to electrode fixation, subjects’ skin was cleaned with an abrasive paste and prepared using an electrode solution (Lubex peeling, Permamed AG, Therwil, Switzerland; Signaspray, Parkerlabs, Fairfield, USA). Male subjects had to be clean shaven. Next, the individualized splints holding the TTFs were firmly attached to the subject’s upper and lower front teeth using a dental composite without etching or bonding (Twinky Star®; VOCO GmbH, Cuxhaven, Germany). Finally, the subject’s head was secured to the headrest of the chair with a strap. This helped to keep the head in an upright position during the experiment and prevented the neck muscles from being activated for stabilization during the jaw closing (Figure 2).

Subjects were carefully instructed to perform two oral tasks. First, they were requested to bite with maximum force on two 10-mm-thick cotton rolls positioned bilaterally on the posterior teeth to record their maximum voluntary contraction (MVC). The resulting muscle activity was used as reference activity [30]. Following this, subjects performed a closing movement against the resistance of an asymmetric external load attached to the left side of the mandible by means of the weight splint. The weights applied were 0 kg, 0.5 kg, 1 kg, 1.5 kg, 2 kg, and again 0 kg. For recordings at 0 kg, participants wore the unloaded weight splint. Each recording began at maximum opening position and ended in maximum intercuspation (i.e., with upper teeth in contact with the weight splint, covering the mandibular teeth). Complete closing phases against the external weight were recorded four times.

### Data Analysis and Statistics

All recordings were stored and analyzed by means of a proprietary software (TMJ-Viewer; EMGAT on Matlab 8.0, The Mathworks, Natick, MA, USA). Data analyses and plots were computed with the statistical software R [31] including the packages *tidyverse* [32], *missForest* [33], and *nparLD* [34]. Jaw-tracking and EMG data were synchronized by means of same-time arrays (same-time steps). Only data recorded during jaw-closing phases were analyzed. The closing phase was defined as the part of the closing movement faster than 5% of its peak velocity.

**Fig. 3:**
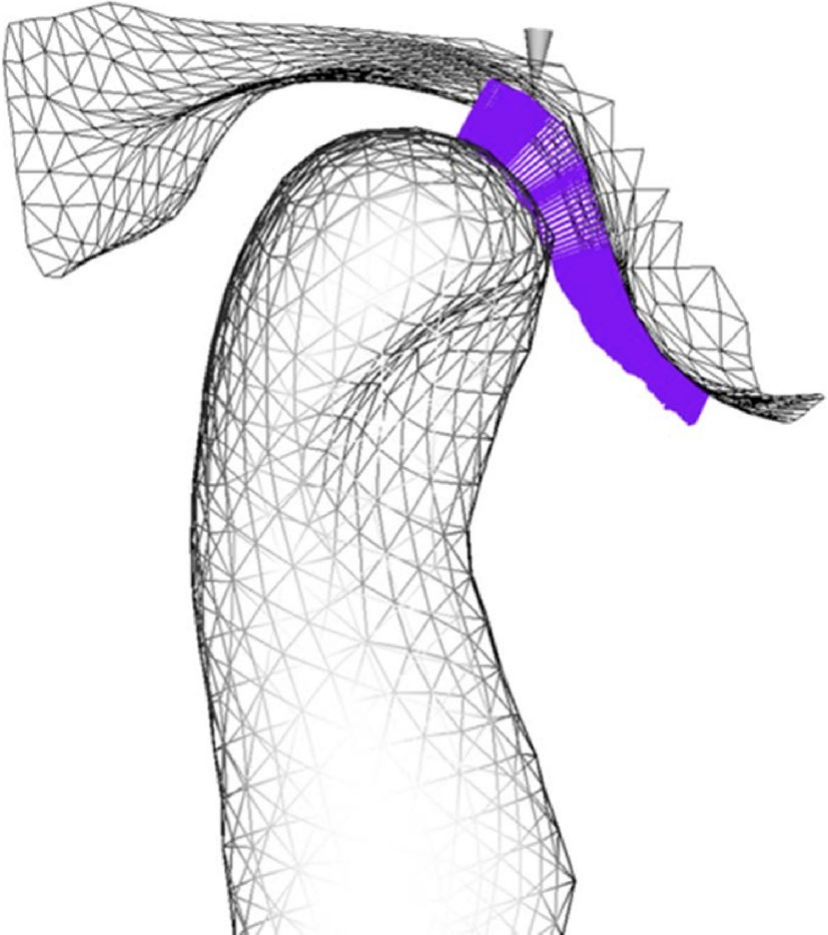
Reconstruction of temporomandibular joint surfaces by triangulation. The violet lines correspond to the minimum distances, calculated for each time step during the closing phase.

To measure the intra-articular space, the value of the minimum intra-articular distance (MID) was calculated at each time step of mandibular motion. MID was defined as the mean of the 30 shortest distances between the triangulation mesh points of the 3D-rendered models of the condyle and fossa, obtained from segmentation of the MR images (Figure 3) [21, 22].

The EMG signal provided a measure of muscle activity. The raw EMG signal was converted to RMS amplitude values (μV) with 60-ms windows and 50% overlap. For inter-individual standardization, RMS values of EMG signal amplitudes were expressed as a % of MVC. (i.e., relative EMG amplitude = EMG_%MVC_, μV/μV). Therefore, changes in the relative EMG amplitude found in the experiment should only be influenced by the loading of the masticatory system. The EMG signal was first analyzed visually (Audacity^©^ package) and then using a proprietary software [27]. For each subject and each recorded movement, bilateral MID and EMG_%MVC_ of the temporalis (EMG_%MVC_ Temp) and masseter (EMG_%MVC_ Mass) muscles were assessed.

The median of the RMS values of EMG_%MVC_ Temp and EMG_%MVC_ Mass during the closing phase were computed for each of these measurements, per subject and movement. Overall summary statistics (mean ± SD) were calculated on the computed median values. Moreover, the difference between the ipsi- and the contralateral side was determined, yielding ΔMID, ΔEMG_%MVC_ Temp, and ΔEMG_%MVC_ Mass, respectively. An increased Δ value therefore means a relatively increased distance or signal from the ipsilateral side. Boxplots and scatterplots were chosen to display the multidimensional dataset and facilitate its interpretation. Local polynomial regression curves show intra-subject trends with increasing weight load (Fig. 5). Moreover, the differences in ΔMID, ΔEMG_%MVC_ Temp, and ΔEMG_%MVC_ Mass with increasing load were analyzed using non-parametric tests for repeated measures [34].

The null-hypotheses tested were that an increasing asymmetric load does not affect the differences between the ipsi- and contralateral side in (i) MID, (ii) EMG_%MVC_ Temp, and (iii) EMG_%MVC_ Mass. The data structure conforms to an LD F1 design and the Wald-Type Statistic was used to globally investigate the influence of the load variable. No pairwise comparisons were computed as such analyses are beyond the scope of the current study. For this inferential analysis, the data were median averaged per subject and load. For the analysis of ΔMID, two subjects were excluded due to some missing values and one value for subject number 7 at load 0.5 kg was input using the MissForest algorithm [33].

## Results

Ten subjects met the inclusion criteria and were included in the study. Two of the ten recruited subjects had to be excluded for the MID analysis, due to a deep bite. For these two subjects, only EMG data were analyzed and MID data were treated as missing. In total, 233 EMG recordings from ten subjects were obtained for the temporalis as well as the masseter muscles. A total of 185 kinematic recordings, from eight subjects, were analyzed for the MID calculation. Two additional subjects were excluded for the inferential analysis of ΔMID (cf. above) and thus, only for this analysis, a total of 144 kinematic recordings were analyzed.

**Fig. 4:**
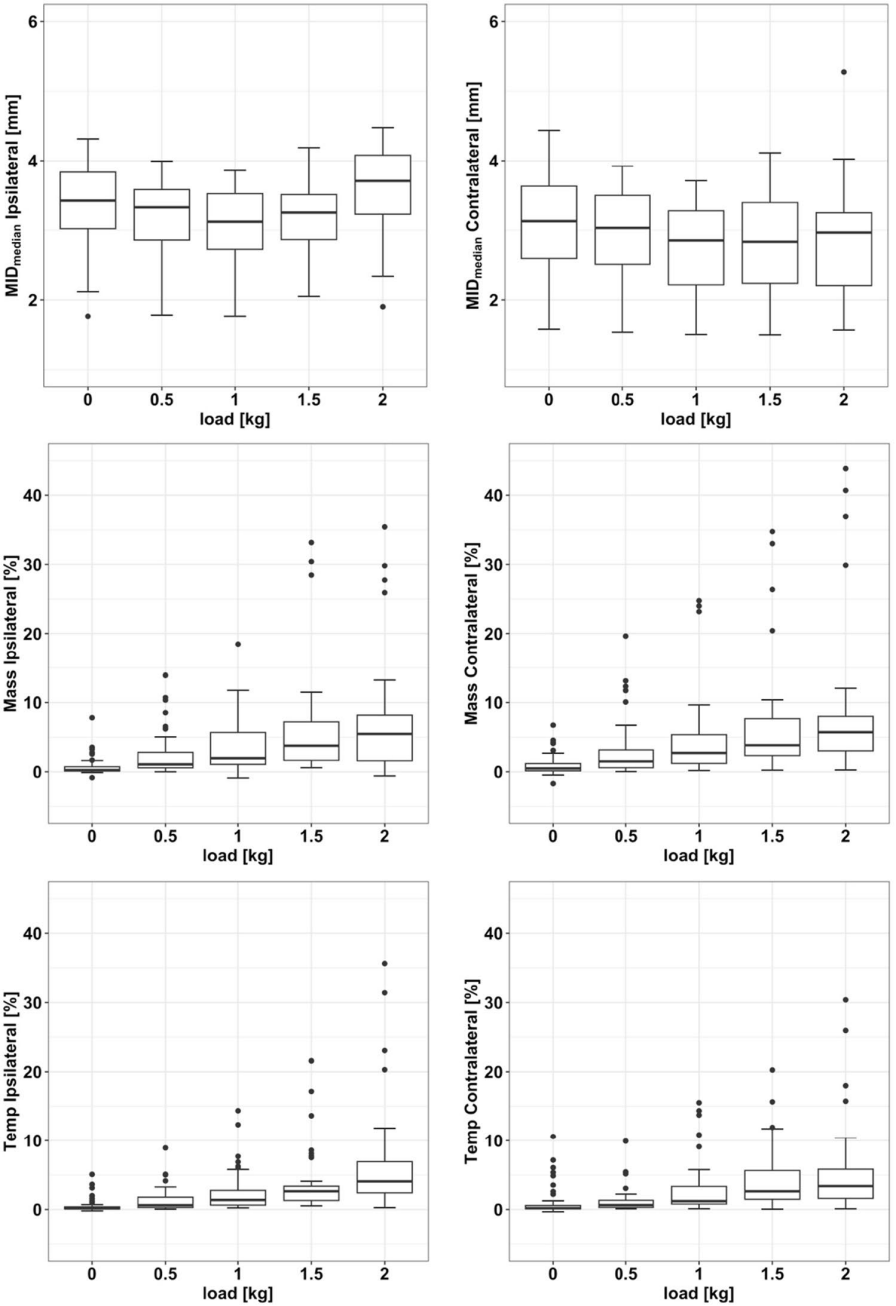
Box plot for MID, EMG_%MVC_ Temp, and EMG_%MVC_ mass. The Y-axis represents the median across all subjects of the minimum distances [mm] in the first line and the EMG_%MVC_ [%] for Mass and Temp in the second and third line, respectively, while the X-axis represents the asymmetrical load applied to the joint [kg].

**Fig. 5:**
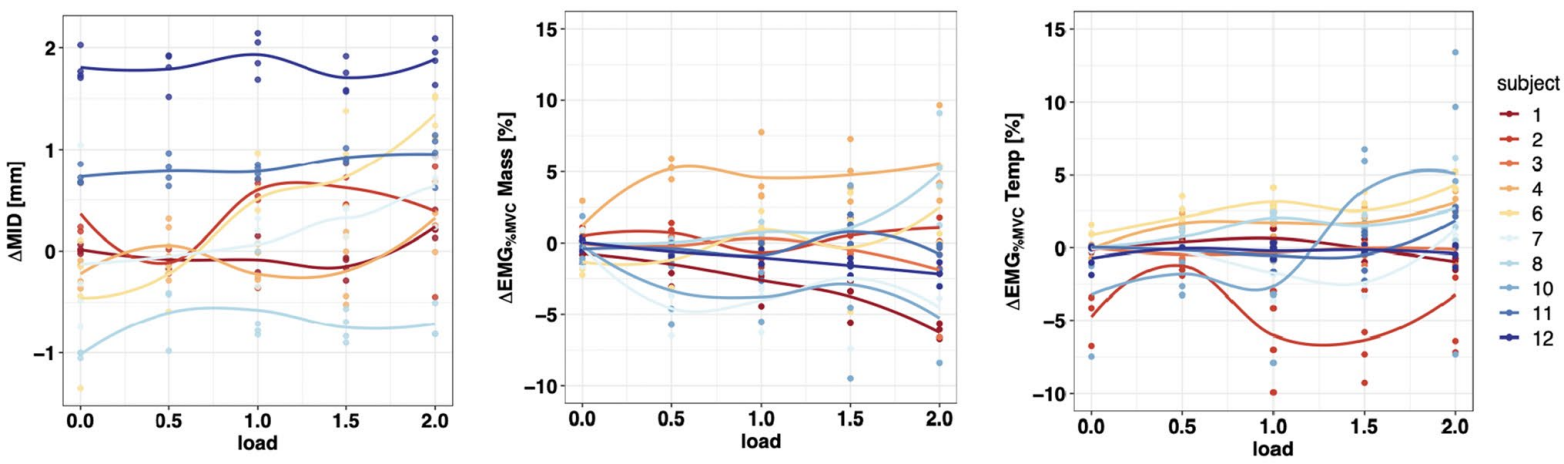
Plots for ΔMID, ΔEMG_%MVC_ Temp, and ΔEMG_%MVC_ Mass per subject and movement. The Y-axis represents the difference between the ipsi- and the contralateral side median of the minimum distances [mm] in the left graphic and for the EMG_%MVC_ [%] in the middle and right graphs, while the X-axis represents the asymmetrical load applied to the joint [kg]. The lines in the graphs are smoothed trendlines using local polynomial regression fits to guide the eye, while the dots represent the actual measurements.

The overall minimum of the MID_median_ was found for both contralateral and ipsilateral joints at 1.0 kg (2.85 mm ± 0.66 and 3.12 mm ± 0.53, respectively). The difference between the minimum and the maximum of the sample-averaged MID_median_ was 0.43 mm for the right joint and 0.48 mm for the left joint. The data boxplot across all subjects can be found in Figure 4. The increase in ΔMID with increasing load was statistically significant (*p* < 0.001). In Figure 5, the local polynomial regression curves show the intra-subject trends in ΔMID with increasing load. Due to anatomic asymmetry, some subjects already showed an ipsilateral-contralateral difference in ΔMID at 0.0 kg. It was found that this difference showed a tendency toward an increase in ΔMID with increasing load attached to the left side during the jaw-closing phase.

For all analyzed subjects, an increase in asymmetric loading of the left mandible led to an increase in the values of EMG_%MVC_ in both the contralateral and the ipsilateral M. temporalis anterior (Fig. 4). Accordingly, the maximum of the sample-averaged EMG_%MVC_ was found for both temporal muscles at 2.0 kg (contralateral: 3.63_%MVC_, ipsilateral: 4.55_%MVC_). This increase in ΔEMG_%MVC_ Temp with increasing load was statistically significant over all subjects (inter-subject comparison, *p* = 0.01). However, there were individual differences between the ipsilateral and contralateral values of EMG_%MVC_ (ΔEMG_%MVC_ Temp) (Fig. 5). Two subgroups with similar activation patterns could be observed: ΔEMG_%MVC_ Temp remained constant or only slightly increased in four subjects (no. 2, 3, 10, 12), whereas this difference clearly increased with increasing load in the other six subjects (no. 1, 4, 6, 7, 8, 11).

Similarly, to the temporal muscle, the EMG_%MVC_ increased in both the contralateral and ipsilateral masseter after increasing the asymmetric load on the mandible (Fig. 4b). The maximum of the sample-averaged EMG_%MVC_ was found for both masseter muscles at 2.0 kg (contralateral: 5.42_%MVC_, ipsilateral: 5.16_%MVC_). However, ΔEMG_%MVC_ Mass with increasing load was not statistically significant across all subjects (inter-subject comparison, *p* = 0.64). Three different patterns could be found when considering the ΔEMG_%MVC_ Mass intra-individually. Four subjects (no. 1, 7, 10, 12) showed a downward trend, whereas three subjects (no. 2, 3, 11) showed constant activity and three subjects (no. 4, 6, 8) showed an upward trend in the difference in EMG activity with increasing loads (Figure 4). Since ΔEMG_%MVC_ Mass showed different intra-individual trends (decreases vs increases vs constant activity).

**Fig. 6:**
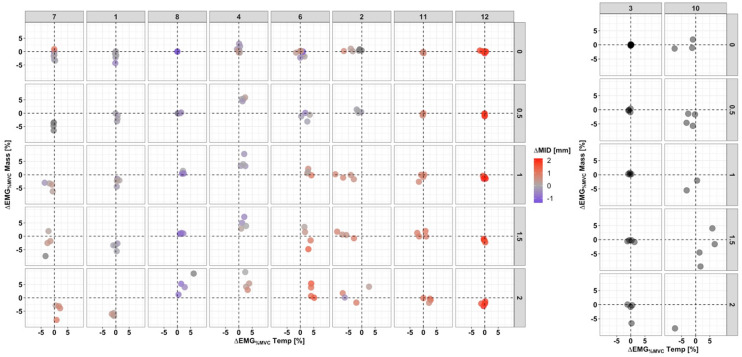
Combined representation of ΔEMG and ΔMID. The Y-axis represents the difference in median relative EMG amplitude [%MVC] of the masseter, while the X-axis represents the difference in median relative EMG amplitude [%MVC] of the anterior temporalis. The difference in the minimum distances is indicated by the size of the points, except for subject no. 3 and no. 10 (missing MID data). *MVC* maximal voluntary contraction.

The multidimensional assessment of the interplay between the variables ΔMID, ΔEMG_%MVC_ Temp, and ΔEMG_%MVC_ Mass is presented in Figure 6. The ΔMID_Median_ for most subjects increased slightly with increasing load. Three different patterns of muscle coordination were observed. Masseter EMG activity became more asymmetric with loading in seven subjects (no. 1, 4, 6, 7, 8, 10, 12): ΔEMG_%MVC_ Mass decreased in four subjects (no. 1, 7, 10, 12), whereas ΔEMG_%MVC_ Mass increased in three subjects (no. 4, 6, 8). Interestingly, these three subjects also showed an increase in ΔEMG_%MVC_ Temp. In the remaining subjects only very moderate changes in ΔEMG_%MVC_ Mass and ΔEMG_%MVC_ Temp were observed, together with a slight increase in ΔMID_Median_.

## Discussion

The main objective of this study was to analyze the effect of asymmetric external loading of the mandible on the minimum intra-articular space and masticatory muscle activation during jaw closure. Dynamic stereometry and electromyography data were acquired in healthy volunteers in order to gain indications on the coordinative strategies of the CNS for mandibular dynamics.

To the authors’ knowledge, this study is the first to analyze the changes in the TMJ intra-articular space during asymmetrically loaded jaw-closing movements, not only three dimensionally and dynamically, but also in combination with electromyographic data of the masticatory muscles. In contrast, other studies only focused on neuromuscular strategies during unilateral and bilateral static biting and only provided insight into isometric situations [4, 5].

First, the results revealed that the asymmetric load produced a decrease in joint space (MID_median_) with a minimum at 1 kg for both joints and a maximum range of 0.43 mm for the contralateral and 0.48 mm for the ipsilateral joint (Fig. 4). This result might indicate an adaptation response of the system throughout the experiment to counteract the stepwise increasing load experienced by both TMJ capsules. One can hypothesize that the asymmetric loading at the beginning of the experiment (0.5–1.0 kg) caused a mechanical disturbance in the system, leading to an increased load in both joints (more on the ipsilateral side). When further load was applied, the perceptual system seemed to react by multiple masticatory muscle activation patterns, resulting in an increase in the MID in the second part of the experiment (1.5–2.0 kg). This might be due to the fact that the load was always applied on the same side and in an increasing manner (from 0 to 2 kg). Therefore, the system could have the possibility to anticipate the application of the next load (feed-forward control mechanism) and masticatory muscles were then activated in a manner to protect the TMJ from overloading. Such behavior was observed in animal models during rhythmic mandibular movements, where the putative feed-forward mechanism was mediated primarily by receptors in the muscle spindles [35, 36]. Thus, this first result might indicate that the CNS pursued a neuromuscular control strategy in asymmetrically loaded closing movements which involve a protective objective, such as MJL, to protect the TMJ from being overloaded. Several mechanisms of joint protection have been proposed in literature. Nickel et al. [4] reported that joint eminence develops in a way that optimizes the direction of condyle loading and thus reduces the overall joint load [37, 38]. Other authors have conducted several further studies to support this hypothesis [5, 39, 40].

Furthermore, the results revealed that the ipsilateral-contralateral difference of the intra-articular space (ΔMID) increased significantly with increasing load. However, this trend seemed to be stronger for some subjects than for others (Figure 5). This might indicate that the CNS, at least in certain subjects, follows a strategy in which the masticatory muscles do not fully compensate for the asymmetric load. In these subjects, it is possible that the principle of MME plays a role.

As seen in Figure 6, the results of muscle activation were more variable. For most subjects (7/10), the asymmetry in the EMG_median_ of the masseter increased with respect to the value at 0 kg. However, it did so with two different trends (downward vs. upward). This contrasts with the anterior temporalis muscle, where a clear increase in the difference of muscle activation was observed for all subjects. It could therefore be assumed that in most subjects the increased asymmetric muscle activation pattern of the anterior temporalis compensated for the asymmetric load. In the others, a combination of masseter and temporalis activation was observed. The inter-individual differences are most likely explained by the different anatomy of the subjects [14] and different muscle activation patterns learned by the subjects during their development. The four masticatory muscles, masseter, temporalis, medial, and lateral pterygoid, together with the suprahyoid muscles, control the complex movement patterns of the mandible in six degrees of freedom [41]. Mandibular movements are the result of a mechanically overdetermined muscular system [42], where several different muscle patterns are possible to produce a particular jaw movement [12, 43]. Bernstein proposed the existence of muscle synergies as a neuronal strategy to simplify the control of multiple degrees of freedom [44]. A functional muscle synergy is defined as a pattern of co-activation of muscles recruited by a single neural command signal [45]. It is assumed that these synergies are formed by each individual through a learning process during development [44]. The stable relationships that eventually become dominant are assumed to have a biological advantage over other possible patterns of muscle activation, such as protecting the joint from overloading. Our results for masseter activation point in the same direction as those of Iwasaki et al. [14]. The Iwasaki group investigated an asymmetric set-up, i.e., unilateral molar biting by means of a computer model based on MME and found, in accordance with some of our results, that the predicted ipsilateral masseter muscle forces were greater than the contralateral ones. They concluded that the mix of muscle and TMJ forces depends on the anatomy of the subject. The high degree of individual variability in the muscular activation patterns could be confirmed in our experiment. Another asymmetrical jaw movement is the masticatory act, during which rhythmic activation is regulated by the central pattern generator of mastication in the brain stem. As shown in previous studies, this movement varies greatly among and within individuals, with age, gender, and with the consistency of the food properties [46, 47].

Schindler et al. [13] found evidence that the medial pterygoid is the most highly recruited muscle relative to all bilateral static biting activities, according to the model used for the calculation. Unfortunately, the study protocol did not include the EMG of the medial pterygoid muscle. It might be argued that in the subjects where the masseter was found to be less active, the ipsilateral medial pterygoid played a role in the muscular compensation of the asymmetrical load.

All experiments of the present study had to be performed with the weight on the same side (left side of the subject), due to the existing technical infrastructure for dynamic stereometry. Therefore, the application of the asymmetrical load could not be side randomized. A further limitation might be the morphological and functional heterogeneity of the examined muscles [48]. Indeed, the activation level of the entire muscle is only approximated by a single recording site. As already reported by Belser et al., the two parts (*pars superficialis*, *pars profunda*) of the masseter are activated to a different extent according to the movement task [49, 50]. Obviously, EMG recordings based on surface electrodes measure the activity of the *pars superficialis* more than the *pars profunda*. Regarding the EMG activity of the temporalis muscle, however, an investigation by Blanksma and van Eijden could not demonstrate any difference in the activity of six different regions within the muscle [51]. In addition, certain intrinsic factors such as joint size, bone thickness, or mechanical properties may also have an influence on the system. In order to minimize the influence of such factors, a homogeneous study population of young and healthy subjects was investigated in this study. In a larger follow-up study, it would be interesting to also measure the intercondylar distance. Depending on the intercondylar distance, the asymmetrically applied load could effectively lead to a slightly different loading of the contralateral joint due to the longer lever arm.

Due to the small sample size and the relative complexity of data collection, caution is required in the interpretation of the study results. Nonetheless, the data and analyses could show a significant effect on MID and EMG_temp_ and thus had sufficient power for these endpoints. This is due to the longitudinal design that does not require a very large sample size but rather repeated measures on the same subject. Furthermore, due to the non-invasive experimental setting, only the EMG activities of superficial masticatory muscles were investigated, omitting the effects of the deep musculature. Nonetheless, the results showed that asymmetrical loads up to 2 kg did affect the intra-articular space in the TMJ.

The data obtained with this experimental setting could be used as input to validate a musculoskeletal model of the masticatory system based on either principle, MJL or MME. This model would be of immediate interest to the field of dentistry and maxillofacial surgery. Such a model could be used to analyze other movements, treatments or surgical procedures in cases where a direct examination of the human TMJ would be too invasive. However, both the MJL and the MME principles could only partially explain the strategy adopted in the studied movement. Despite the sample size, the present results suggest that when the system is confronted with an unexpected load during the closing movement, then a control mechanism, which has to be learned in the course of repetitive movements, might be involved.

In conclusion, the MID and EMG of the anterior temporalis were affected by the application of an asymmetrical load during a symmetric jaw-closing movement. Indications were gained that an asymmetrically loaded jaw-closing movement might involve a control mechanism protecting the joints from overloading. However, this mechanism needed to be learned in the course of the experiment. This protection against asymmetric loading involved muscular compensation, especially an increase in the ipsilateral-contralateral difference in the activation of the muscle temporalis anterior. Thus, the results do not fully support the hypothesis of MJL nor MME strategy.
